# Use of Ensure® nutrition shakes as an alternative formulation method for live recombinant Attenuated *Salmonella* Typhi vaccines

**DOI:** 10.1186/s12866-015-0409-5

**Published:** 2015-03-29

**Authors:** Karen E Brenneman, Amanda Gonzales, Kenneth L Roland, Roy Curtiss

**Affiliations:** The Biodesign Institute, Arizona State University, Tempe, AZ 85287 USA; School of Life Sciences, Arizona State University, Tempe, AZ 85287 USA; Present address - 23andMe, Inc, 1390 Shorebird Way, Mountain View, CA 94043 USA

**Keywords:** *Salmonella* vaccine, Gastric pH neutralization, Bicarbonate, Ensure nutrition shake, Low gastric pH mouse model

## Abstract

**Background:**

To be effective, orally administered live *Salmonella* vaccines must first survive their encounter with the low pH environment of the stomach. To enhance survival, an antacid is often given to neutralize the acidic environment of the stomach just prior to or concomitant with administration of the vaccine. One drawback of this approach, from the perspective of the clinical trial volunteer, is that the taste of a bicarbonate-based acid neutralization system can be unpleasant. Thus, we explored an alternative method that would be at least as effective as bicarbonate and with a potentially more acceptable taste. Because ingestion of protein can rapidly buffer stomach pH, we examined the possibility that the protein-rich Ensure® Nutrition shakes would be effective alternatives to bicarbonate.

**Results:**

We tested one *Salmonella enterica* serovar Typhimurium and three *Salmonella* Typhi vaccine strains and found that all strains survived equally well when incubated in either Ensure® or bicarbonate. In a low gastric pH mouse model, Ensure® worked as well or better than bicarbonate to enhance survival through the intestinal tract, although neither agent enhanced the survival of the *S*. Typhi test strain possessing a *rpoS* mutation.

**Conclusions:**

Our data show that a protein-rich drink such as Ensure® Nutrition shakes can serve as an alternative to bicarbonate for reducing gastric pH prior to administration of a live *Salmonella* vaccine.

## Background

Live recombinant attenuated *Salmonella*-vectored vaccines (RASV) have the potential to provide protection against a variety of human non-*Salmonella* pathogens at low cost. By using the *Salmonella* cells to express a heterologous protective antigen, RASVs can induce humoral and cellular immune responses directed at a pathogen of interest [1]. RASVs have the additional advantage of stimulating mucosal immune responses, due to their oral route of immunization. Oral delivery provides RASVs with the opportunity to invade and colonize the intestinal gut-associated lymphoid tissues (GALT), where they actively interact with the host immune system to stimulate robust humoral, mucosal and cellular immune responses [2]. To allow the vaccine cells to reach the intestinal tissues more rapidly, human subjects are frequently required to fast prior to immunization as a means to clear the gastrointestinal tract of food [3]. However, fasting also causes the gastric pH of humans to fall below 2.0 [4,5]. This poses a non-trivial challenge to the success of the immunization, as *Salmonella* species, particularly *S*. Typhi [6], are not particularly resistant to low pH (succumbing below pH 3.0), and the mutations necessary for attenuation in RASVs often impose additional sensitivity to acid [7-11]. Our lab has constructed RASV strains exhibiting regulated-delayed attenuation [12]. These *S*. Typhi-derived RASVs, χ9633(pYA4088), χ9639(pYA4088) and χ9640(pYA4088), are susceptible to a number of environmental stresses, including low pH [13]. To administer an acid-sensitive vaccine strain via the oral route, the vaccine must be given using a strategy that not only actively protects the vaccine cells from gastric acid, but also does not negatively affect vaccine viability or the development of an immune response following vaccination.

Most researchers address the problem of low gastric pH by administering an antacid such as sodium bicarbonate prior to the RASV [14-17]. The antacid rapidly neutralizes the gastric acid, allowing the vaccine cells to transit the gastric compartment under neutral or mildly acidic conditions [18,19]. This combination of a liquid RASV formulation with antacid is highly effective and promotes the development of protective immune responses [20,21]. However, bicarbonate is not without problems. In order to efficiently neutralize gastric acid, a surprisingly large volume of bicarbonate must be given, as gastric mixing is not efficient enough to thoroughly disperse small volumes of bicarbonate completely throughout the stomach [22,23]. In addition, bicarbonate has a rather unpleasant taste to most palates and efforts to improve this aspect will positively enhance the experience of the volunteers in a clinical trial or vaccinees receiving licensed vaccines. Flavoring agents are sometimes added to vaccine formulations for this reason [24].

In preparation for a clinical trial to assess the three RASV strains listed above, we wanted to investigate the administration of high concentrations of protein as an alternative to bicarbonate. Protein is capable of buffering gastric acid and raises the gastric pH within minutes of ingestion [25,26]. As a food-borne pathogen, *Salmonella* appears to take advantage of this gastric acid buffering during infection scenarios. In the presence of protein-rich food, the infectious dose of *Salmonella* is significantly lower than in the absence of food [27]. Thus, we hypothesized that the administration of protein, specifically Ensure® Nutrition shakes, immediately prior to and following immunization would provide the same protection from the low pH gastric environment as bicarbonate. Using Ensure® also provides a carrier with a taste likely to be more pleasant than bicarbonate for most vaccinees. We examined the survival of *S*. Typhi wild type and vaccine strains when suspended in Ensure® or a bicarbonate solution and how these compounds, when administered to mice with a low gastric pH, influenced survival during gastric transit.

## Results

### Survival of recombinant attenuated *Salmonella* Typhi vaccine strains in bicarbonate and Ensure®

**Figure 1:**
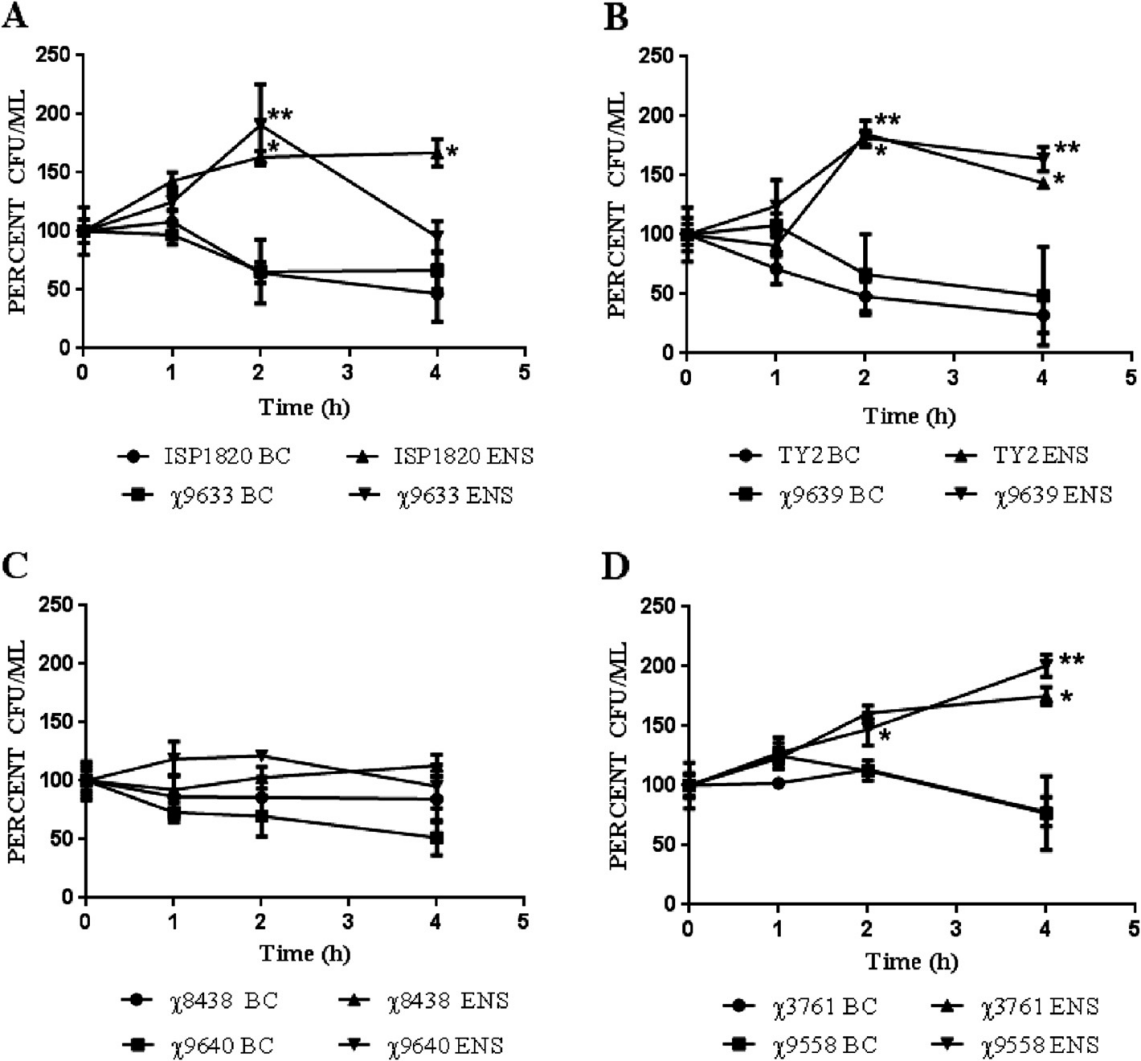
**Survival of** ***Salmonella*** **vaccine and wild-type parent strains in Ensure and sodium bicarbonate. (A)** *S*. Typhi strains χ9633 and χ3744; **(B)** *S*. Typhi strains χ9639 and χ3769; **(C)** *S*. Typhi strains χ9640 and χ8438; **(D)** *S*. Typhimurium χ9558 and χ3761. Percent CFU/ml was calculated as follows: (# CFU at t_n_/# CFU at t_0_) x 100 for each strain. Wild-type strains that exhibited a significant increase (*p* < 0.05) in the number of viable cells in Ensure over viable cells in bicarbonate are marked with an asterisk (*). Vaccine strains exhibiting a significant increase in viable cells (*p <* 0.05) between Bicarbonate and Ensure treatments are marked with a double asterisk (**). Data are the combined results of three independent experiments.

To be an effective vaccine formulation, the carrier or co-administered substance must not negatively affect the viability of the vaccine cells. We monitored the effect of bicarbonate and Ensure® (milk chocolate flavor) on the viability of the three *S.* Typhi vaccine strains and model *S.* Typhimurium strain for four hours (Figure 1). Most of the *S*. Typhi strains, including χ9633, χ9639, ISP1820 and Ty2, and both of the *S*. Typhimurium strains we tested underwent a statistically significant increase in numbers when incubated in Ensure®, indicating that Ensure® could support the growth of these strains. Cell numbers of strains suspended in bicarbonate tended to decrease over time, but the decrease was statistically significant only for strain Ty2 (T_0_ vs T_4_, *p* = 0.035) (Figure 1B). Interestingly, there were no significant changes in cell numbers for strains χ8438 and χ9640 in either bicarbonate or Ensure® (Figure 1C). There were statistical differences in the numbers of cells recovered from Ensure compared to bicarbonate at the 2 and 4 h time points for a number of strains (Figure 1A, B, D), primarily due to the fact that Ensure® apparently supported the growth of these strains while bicarbonate did not. We also examined survival in vanilla and strawberry Ensure® and the flavor did not affect strain viability (data not shown).

### Bicarbonate and Ensure® protect vaccine cells during low pH gastric transit

**Figure 2:**
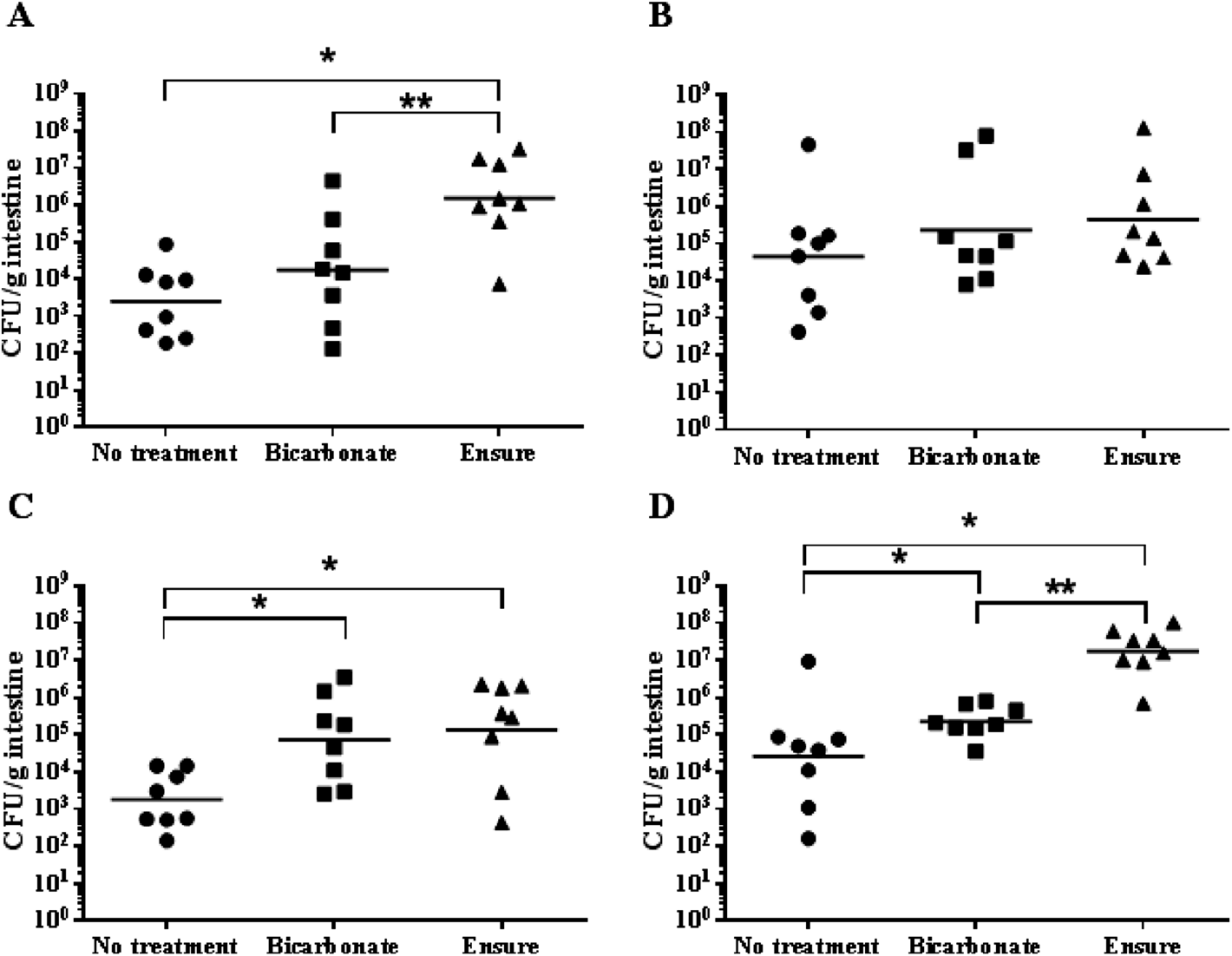
**Survival of RASV strains during low pH gastric transit.** Histamine-treated mice were inoculated orally with 10^9^ CFU of **(A)** χ9633(pYA4088, pWSK129), **(B)** χ9639(pYA4088, pWSK129), **(C)** χ9640(pYA4088, pWSK129) or **(D)** χ9558(pYA4088, pWSK129). Mice received either 1.3% sodium bicarbonate, chocolate Ensure® or no treatment prior to and immediately following immunization to neutralize gastric acid. The number of viable vaccine cells in the small intestine was quantified one hour after immunization. Data are presented as the number of CFU/g intestine of individual mice, with the geometric mean of the group displayed as a solid horizontal line. Groups that exhibited a significant increase (*p* < 0.05) in the number of viable vaccine cells in the small intestine over the control group are marked with an asterisk (*). Groups exhibiting a significant difference (*p <* 0.05) between Bicarbonate and Ensure treatments are marked with a double asterisk (**). Data are the combined results of two independent experiments (8 mice total).

Another characteristic of an effective RASV delivery formulation is that it must protect cells from the low pH of the gastric environment. To examine the ability of bicarbonate and Ensure® to combat gastric pH, these were used to buffer the stomach pH of mice. Because the gastric pH of a fasted mouse is about pH 4.0 and the gastric pH of a fasted human is about pH 1-2 [4,5,28], gastric acid secretion was induced in mice by subcutaneous histamine injection (see Methods section) prior to immunization to better mimic the situation in humans. Using this protocol, the pH in the mouse stomach is reduced to around 1.5 [29]. Mice received either bicarbonate or Ensure® prior to and immediately following immunization. Control mice received no treatment. Vaccine viability was measured following gastric transit (Figure 2). Compared to the no treatment group, administration of Ensure® significantly increased the number of viable cells that reached the small intestine for two of the *S*. Typhi strains and for the *S*. Typhimurium strain (*p* = 0.0019 for χ9633(pYA4088), *p* = 0.0256 for χ9640(pY4088) and *p* = 0.0006 for χ9558(pYA4088). This was a 599-, 75.0- and 647-fold increase, respectively, in the geometric mean number of viable cells to reach the ileum. Bicarbonate similarly improved the survival of χ9640(pYA4088) (*p* = 0.0190) and χ9558(pYA4088) (*p* = 0.0379) during gastric transit, resulting in a 41.0- and 8.79-fold increase in the geometric mean number of cells to reach the ileum, respectively. Administration of bicarbonate did not significantly impact the survival of χ9633(pYA4088) or χ9639(pYA4088) (*p* = 0.2317 and 0.4945, respectively) compared to the no treatment controls.

The Ensure treatment was better than bicarbonate at increasing the gastric transit survival of strains χ9633(pYA4088) (*p* = 0.0207) and χ9558(pYA4088) (*p* = 0.0003). Interestingly, the survival of strain χ9639(pYA4088) was not impacted by either Ensure or bicarbonate treatments (Figure 2B). Further, this strain survived gastric transit in mice that did not receive bicarbonate or Ensure® somewhat better than the other *S*. Typhi strains (Figure 2A, B, C) although the difference was not statistically significant (*p* = 0.06).

## Discussion

The vast majority of clinical RASV trials have made use of sodium bicarbonate as a means to protect vaccine cells from low gastric pH. In fact, field trials with the licensed typhoid vaccine strain Ty21a demonstrated that the administration of bicarbonate produced a superior immune response as compared to other vaccine formulation strategies [30,31]. Our results are consistent with the idea that ingestion of a buffering substance prior to oral immunization promotes the survival of vaccine cells. The administration of bicarbonate prior to and immediately following immunization significantly improved the survival of both *S.* Typhi χ9640(pYA4088) and *S.* Typhimurium χ9558(pYA4088) during gastric transit. Interestingly, strain χ9640(pYA4088) was the most immunogenic, among the three *S*. Typhi strains tested here, in a recent clinical trial [32]. Sodium bicarbonate is generally regarded as safe, and has been shown to have no effect on the viability of wild-type *Salmonella* [33].

Our results demonstrated that high concentrations of protein administered before and after immunization can act as a substitute for bicarbonate. Ensure® provided a greater degree of protection from the gastric environment than bicarbonate for *S*. Typhi strain χ9633(pYA4088, pWSK129) (Figure 2A) and *S*. Typhimurium strain χ9558(pYA4088, pWSK129) (Figure 2D), and provided protection equivalent to bicarbonate for *S*. Typhi strain χ9640(pYA4088, pWSK129) (Figure 2C). No effect of bicarbonate or Ensure® was observed for the *rpoS* Ty2 derivative, *S*. Typhi strain χ9639(pYA4088, pWSK129) (Figure 2B).

Neither sodium bicarbonate nor Ensure® was able to significantly increase the survival of χ9639(pYA4088) during gastric transit. This is interesting, because of the four RASV strains tested in this study, χ9639 is the only *rpoS* mutant, due to the fact that parent strain Ty2 carries a mutation in *rpoS* [34]. *Salmonella rpoS* mutants are significantly more sensitive to low pH than strains with a functional RpoS because they are unable to sustain an acid tolerance response (responsible for protecting cells against low pH) for more than 20 minutes [7]. The problem may have been exacerbated by the presence of the ΔP_fur81_::TT *araC* P_BAD_*fur* mutation in χ9639, as Fur and RpoS jointly regulate induction of the acid tolerance response [7,35]. The amount of Fur present in a ΔP_fur81_::TT *araC* P_BAD_*fur S*. Typhi mutant is substantially lower than a wild-type strain, regardless of the arabinose concentration during growth and, with regard to survival at low pH, is indistinguishable from a *fur* deletion mutant (29). Note that strain χ9640 is also a derivative of Ty2, but in this strain, the *rpoS* gene has been replaced with a functional gene from ISP1820 (parent of χ9633). The S. Typhimurium strain χ9558 has a functional *rpoS* gene, since its parent is RpoS^+^. Thus, it is likely that a functional *rpoS* is required in order to benefit from bicarbonate and Ensure treatment, at least in this genetic background.

## Conclusions

The Ensure® nutrition shake was able to act as a substitute for bicarbonate during oral inoculation to enhance bacterial survival during passage through a low gastric pH compartment. Ensure® provided protection better than or equivalent to bicarbonate for all of the strains tested. The failure of both Ensure® and bicarbonate to protect an *rpoS* mutant during gastric transit suggests that in future clinical trials, investigators should carefully evaluate the degree of protection necessary for the specific RASV strain being evaluated and perform a careful evaluation of the buffering agent used to neutralize gastric pH.

## Methods

### Bacterial strains, plasmids and culture conditions

The bacterial strains and plasmids used in this study are listed in Table 1. Strain χ9633 is derived from *S*. Typhi ISP1820, an RpoS^+^ strain. Strains χ9639 and χ9640 are derived from parent strain Ty2, which is RpoS^−^. Strain χ9640 was rendered RpoS^+^ by transduction [13]. For routine use, strains were propagated in LB medium (which contains 0.1% glucose) [36] supplemented with 0.05% arabinose and 0.1% mannose at 37°C. Some experiments included KT broth, which is a proprietary medium used to support rapid, high-density bacterial growth, similar in composition to terrific broth [13]. For antibiotic selection of strains containing pWSK129, kanamycin was used at a concentration of 30 μg/ml. All chemicals were purchased from Sigma-Aldrich (St. Louis, MO, USA) or Thermo Fisher Scientific (Pittsburgh, PA, USA) unless otherwise indicated.

**Table 1:** ***Salmonella*** **vaccine strains and plasmids used in this study**

**Strain**	***Salmonella*** **Serovar**	**Genotype/Phenotype** ^**a**^	**Reference**
χ9558	Typhimurium	∆*pmi-2426* ∆(*gmd-fcl*)*-26* ∆P_fur81_::TT *araC* P_BAD_ *fur* ∆P_crp527_::TT *araC* P_BAD_ *crp* ∆*asdA27*::TT *araC* P_BAD_ *c2* ∆*araE25* ∆*araBAD23* ∆*relA198*::*araC* P_BAD_ *lacI* TT ∆*sopB1925* ∆*agfBAC811*, RpoS^+^	[39]
χ9633	Typhi ISP1820	∆P_crp527_::TT *araC* P_BAD_ *crp* ∆P_fur81_::TT *araC* P_BAD_ *fur* ∆*pmi-2426* ∆(*gmd-fcl*)*-26* ∆*relA198*::*araC* P_BAD_ *lacI* TT ∆*araE25* ∆*araBAD23* ∆*tviABCDE10* ∆*agfBAC811* ∆*sopB1925* ∆*asdA33*, RpoS^+^	[13]
χ9639	Typhi Ty2	∆P_crp527_::TT *araC* P_BAD_ *crp* ∆P_fur81_::TT *araC* P_BAD_ *fur* ∆*pmi-2426* ∆(*gmd-fcl*)-26 ∆*relA198*::*araC* P_BAD_ *lacI* TT ∆*araE25* ∆*tviABCDE10* ∆*agfBAC811* ∆*sopB1925* ∆*asdA33*, RpoS^−^	[13]
χ9640	Typhi Ty2	∆P_crp527_::TT *ara*C P_BAD_ *crp* ∆P_fur81_::TT *araC* P_BAD_ *fur* ∆*pmi-2426* ∆(*gmd-fcl)-26* ∆*relA198*::*ara*C P_BAD_ *lacI* TT ∆*araE25* ∆*tviABCDE10* ∆*agfBAC811* ∆*sopB1925* ∆*asdA33*, RpoS^+^	[13]
χ3761	Typhimurium	wild type	[40]
χ3744	Typhi	ISP1820 wild type	[41]
χ3769	Typhi	Ty2 *rpoS*	[42]
χ8438	Typhi	Ty2 RpoS^+^	[43]
**Plasmid**		**Description** ^**b**^	
pWSK129		pSC101 *ori*, Kan^r^	[44]
pYA3493		pBR *ori*, Asd^+^ vector with *bla* SS-based periplasmic antigen secretion	[45]
pYA4088		Encodes the α-helical region of PspA (aa 3-285) in pYA3493	[46]

^a^In genotype descriptions, the subscripted number refers to a composite deletion and insertion of the indicated gene. P, promoter; TT, T4 ip III transcription terminator.

^b^ *ori*, replication of origin; SS, secretion signal; Kan^r^, kanamycin resistance.

### Formulation stability assays

Strains were grown in KT broth to an optical density at 600 nm of 2.0, then were pelleted and resuspended in phosphate buffered saline (PBS) at 5 x 10^10^ CFU/ml. Cells were diluted 1:15 into either a 1.3% sodium bicarbonate solution or Ensure® Nutrition shake (milk chocolate flavor) and incubated at 37°C for four hours. Viability at each time point was assessed by serial dilution and plating onto LB agar containing 0.2% arabinose.

### Gastric transit assays

This study was approved by the Arizona State University Institutional Animal Care and Use Committee. Six week old, female BALB/c mice (Charles River Laboratories, Wilmington, MA, USA) were fasted without food or water for 6 h prior to the start of the experiment. Mice received the histamine H_1_-receptor antagonist chlorpheniramine (0.3 mg/kg) subcutaneously to prevent allergy/anaphylaxis symptoms. Prior to inoculation, low gastric pH was induced by subcutaneous injection of histamine dihydrochloride (10 mg/kg) [37,38]. All bacterial strains used in the gastric transit assays contained the low copy number plasmid pWSK129 (Kan^r^) to allow for precise quantitation of strain numbers in the non-sterile environment of the gastrointestinal tract. We did not observe any Kan^r^ organisms in the normal intestinal flora of the mice. Strains were grown to late log phase (optical density at 600 nm of 0.9), then pelleted and resuspended in PBS at a concentration of 5 x 10^10^ CFU/ml. Groups of 5 mice were orally inoculated 50 min after the administration of histamine [29]. For each inoculation, the low gastric pH was treated with sodium bicarbonate, Ensure, or left untreated. Groups that were treated with bicarbonate received 40 μl of a 1.3% sodium bicarbonate solution orally 10 minutes prior to inoculation and an additional 10 μl 10 minutes after [17]. Groups that were treated with Ensure received 20 μl of Ensure® Nutrition shake (milk chocolate flavor) 10 minutes prior to inoculation and an additional 20 μl 10 minutes after [32]. Mice were euthanized 1 h after inoculation and the entire small intestine was removed, homogenized and serially diluted. Samples were plated onto LB agar containing 0.2% arabinose with kanamycin to determine the number of viable bacteria present following low pH gastric transit.

### Statistical analyses

All statistical analyses were performed using GraphPad Prism version 6.00 for Windows (GraphPad Software, La Jolla California USA). Statistical analyses of data from the gastric transit assays were performed using the Mann-Whitney test. Survival curves were analyzed using Sidak’s multiple comparison test.

## Abbreviations

PBS: Phosphate buffered saline
Kan^r^: Resistant to the antibiotic kanamycin
